# Integrated conservation of linear heritage landscapes from a biocultural diversity perspective: A case study of the Huizhou-Hangzhou Ancient Trail, China

**DOI:** 10.1371/journal.pone.0360922

**Published:** 2026-10-22

**Authors:** Yuanliang Dong, Chengcheng Pan, Siyu Pan, Lei Zhang

**Affiliations:** College of Civil and Architectural Engineering, Chuzhou University, Chuzhou, China; Zhejiang Agriculture and Forestry University: Zhejiang A and F University, CHINA

## Abstract

Linear cultural heritage landscapes embody the long-term coupling relationships between ecological processes and human cultural practices, yet conservation models guided by a single ecological or cultural goal fail to meet their holistic management needs. Based on the perspective of biocultural diversity, this study takes the Huizhou-Hangzhou Ancient Trail (HHAT) as a case study and constructs a comprehensive conservation framework oriented toward spatial planning. Biodiversity and cultural diversity patterns are assessed and their spatial relationships are quantified using the coupling coordination degree model. The biocultural diversity index is further integrated with the Marxan systematic conservation planning model to identify Biocultural Core Protected Areas (BCCPA) and support differentiated spatial conservation strategies. The results indicate that biodiversity and cultural diversity along the HHAT exhibit a pronounced antagonistic coupling relationship: regions with antagonistic coupling account for 98.90% of the total study area, while coordinated regions make up only 0.02%. Based on the natural and cultural context of the study area, this pattern may be jointly influenced by rigid competition for small‑scale spatial resources, the dual effects of traffic corridors, and the cumulative effects of traditional livelihood activities. In response, an integrated conservation spatial pattern of one core area and two corridors is proposed, together with four conservation strategies: Conservation-Minimal Intervention, Conservation-Appropriate Revitalization, Restoration-Minimal Intervention, and Restoration-Appropriate Revitalization-enabling an effective translation from coupling diagnosis to spatial planning responses. This study advances understanding of biocultural interactions in mountainous linear heritage landscapes and provides a transferable methodological pathway for integrated conservation planning.

## 1 Introduction

Against the backdrop of globalization and rapid urbanization, the conservation paradigm of cultural heritage has shifted from individual conservation to integrated conservation, and further evolved toward sustainable conservation [1]. Its scope has expanded from individual heritage items to the landscape scale, encompassing a more comprehensive range of heritage values [2]. In particular, Linear Cultural Heritage—such as ancient trails and canals—owing to their cross-regional linear or belt-like attributes, integrate with natural environments, settlement patterns, and socio-cultural practices to form a comprehensive cultural landscape system [3]. These heritage sites are not only a testament to the interconnection of human civilizations, but also serve as vital corridors for maintaining regional ecological connectivity [4]. However, the existing one-dimensional conservation approaches focusing solely on either ecological or cultural aspects pose severe challenges to the landscape integrity of linear cultural heritage, including disrupted spatial continuity, impeded ecological processes, and diminished cultural connectivity [4,5]. The Biocultural Diversity Theory posits that biodiversity and cultural diversity are inherent and indivisible linked [6]. For ancient trails, human commercial activities and settlement evolution are closely coupled with local topography, vegetation, and ecological succession, forming a typical socio-ecological system [7]. In recent years, the International Union for Conservation of Nature (IUCN) has emphasized that integrating a biocultural perspective constitutes a key pathway to achieving landscape resilience and sustainable development [8]. Therefore, developing a comprehensive conservation framework that integrates biological and cultural diversity is pivotal to the long-term sustainable management of linear cultural heritage landscapes.

In existing studies, GIS spatial analysis and the Minimum Cumulative Resistance (MCR) model have been widely adopted to reveal the suitability pattern of heritage corridors under the dual constraints of natural environments and human activities [9,10]. Meanwhile, some studies have introduced the graph theory analysis framework, which identifies and evaluates the topological structure, connectivity and key nodes of the heritage corridor system by constructing cultural heritage node-corridor networks, thus expanding the structural perspective of heritage corridor research [11]. On this basis, the application of circuit theory has enabled a comprehensive characterization of potential heritage transmission paths, and can spatially identify the key passages and bottleneck areas within corridor networks, thereby providing more detailed support for the risk assessment of heritage corridor fragmentation and the formulation of conservation priorities [12,13]. Furthermore, some studies have proposed frameworks for the construction of regional-scale cultural-ecological corridors, attempting to coordinate the objectives of cultural heritage conservation, ecological security patterns and territorial spatial planning at a more macro scale, thus providing theoretical support for the cross-regional integrated conservation of linear cultural heritage landscapes [14,15]. Generally, existing research suffers from inherent limitations. For one thing, most studies isolate cultural values from ecological functions as discrete evaluation dimensions, lacking a systematic characterization of their coupling mechanism and associated spatial heterogeneity. For another, they are deficient in the quantitative optimization of conservation objectives, the construction of scenario-based decision support systems, and the formulation of dynamic governance strategies, which hiders the in-depth implementation of comprehensive conservation for linear cultural heritage landscapes. In addition, research on linear cultural heritage landscapes has focused primarily on large-scale linear heritage sites such as the Silk Road [16] and the Great Wall [17], while insufficient attention has been paid to small-scale regional linear heritage sites represented by She County and Jixi County [18].

Based on the aforementioned research background and gaps, this study takes the Huizhou-Hangzhou Ancient Trail (HHAT) in China as its subject. It aims to construct an integrated spatial conservation framework for biocultural diversity tailored to linear cultural heritage landscapes. This framework explicitly maps assessments of biological and cultural values to specific geographic units and trail segments, providing quantitative evidence for conservation zoning and management decisions. The main contributions of this study include: (1) exploring the coupling relationship between biological and cultural diversity and their spatial heterogeneity; (2) integrating biocultural diversity indices with Marxan model analysis to offer an operational analytical framework for the synergistic conservation of biocultural heritage landscapes.

## 2 Study area and data sources

### 2.1 Study area

The HHAT refers to a system of multiple ancient trails historically connecting the ancient Huizhou Prefecture and Hangzhou Prefecture (Table 1), covering 12 townships and towns including She County of Huangshan City and Jixi County of Xuancheng City (Fig 1). Over the long course of history, the HHAT area has served as a vital conduit for commercial exchanges, population migration, and cultural interactions between the inland southern Anhui region and the coastal areas of Zhejiang Province, fostering a highly systematic linear cultural heritage landscape.

**Table 1:** Main components and conservation status of the HHAT.

Trail type	Trail name	Present status
Inter-prefectural Ancient Trail	Dengyuan Ancient Trail (Dengyuan AT)	Reconstructed
	Huichang Ancient Trail (Huichang AT)	Reconstructed
	Xiaoyaoling Ancient Trail (Xiaoyaoling AT)	Preserved
	Laozhuling Ancient Trail (Laozhuling AT)	Preserved
Inter-county Ancient Trail	Huiqing Ancient Trail (Huiqing AT)	Reconstructed
	Congshanguan Ancient Trail (Congshanguan AT)	Reconstructed
	Huifu Ancient Trail (Huifu AT)	Reconstructed
	Bancha Ancient Trail (Bancha AT)	Reconstructed
	Shuizhukeng Ancient Trail (Shuizhukeng AT)	Reconstructed
	Wangkengling Ancient Trail (Wangkengling AT)	Preserved
	Huanlianling Ancient Trail (Huanlianling AT)	Preserved
Rural Ancient Trail	Yangxi-Fuling Ancient Trail (Yangxi-Fuling AT)	Preserved
	Jingkanling Ancient Trail (Jingkanling AT)	Preserved
	Zhuling Ancient Trail (Zhuling AT)	Preserved
	Changgangling Ancient Trail (Changgangling AT)	Preserved
	Yunling Ancient Trail (Yunling AT)	Preserved
	Songyantang Ancient Trail (Songyantang AT)	Preserved
	Fenggaoling Ancient Trail (Fenggaoling AT)	Preserved
	Dazhang Ancient Trail (Dazhang AT)	Reconstructed
	Fuling-Jiapeng Ancient Trail (Fuling-Jiapeng AT)	Reconstructed
	Dingling Ancient Trail (Dingling AT)	Reconstructed
	Dade Ancient Trail (Dade AT)	Preserved
	Jinsha-Jiapeng Ancient Trail (Jinsha-Jiapeng AT)	Reconstructed
	Huiling Ancient Trail (Huiling AT)	Preserved
	Jinzhou Ancient Trail (Jinzhou AT)	Reconstructed
	Gexiyuan Ancient Trail (Gexiyuan AT)	Reconstructed

*HHAT: Huizhou-Hangzhou Ancient Trail; AT: Ancient Trail.

**Fig 1:**
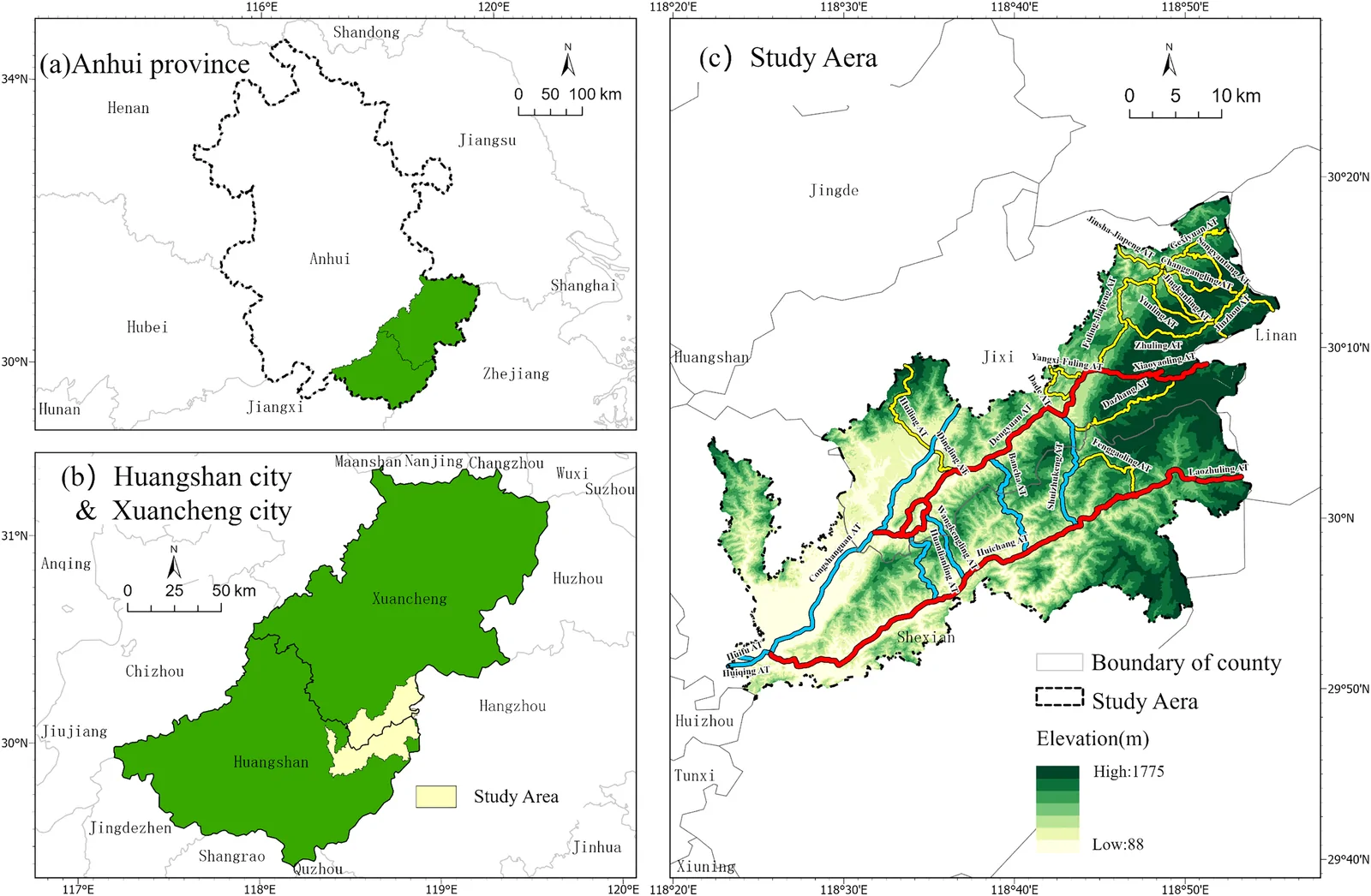
Location of the HHAT study area. HHAT: Huizhou-Hangzhou Ancient Trail. Note: This figure was created using map data from Tianditu, available at http://www.tianditu.gov.cn/.

To ensure the consistency and comparability of multi-source landscape, land use, and cultural heritage data, township administrative boundaries were adopted to define the overall study extent rather than acting as the basic analytical units. This delineation preserves the integrity of the HHAT cultural system—comprising ancient trails, historic villages, cultural relics, and intangible heritage registered at the township level. An alternative fixed-width buffer along the trail corridor was considered, but is better suited for nature-focused studies such as biodiversity assessment. For this integrated bio-cultural study, a buffer would exclude culturally linked settlements and heritage sites outside the buffer zone, compromising the integrity of the cultural system. This boundary choice has limitations: modern administrative boundaries do not perfectly align with the naturally extended scope of the historical trail corridor, which may introduce slight statistical biases in coupling pattern analysis. We prioritized the integrity of cultural heritage and data availability, and selected townships intersected by the HHAT as the study area. It covers Huicheng, Xiakeng, Bei’an, Guilin, Qizili, Sanyang in She County, and Huayang, Linxi, Yingzhou, Fuling, Jiapeng, Jingzhou in Jixi County, totaling approximately 1,268.94 km^2^.

The HHAT area is located in the core area of the Southern Anhui Mountainous Region, serving as the geographical transition zone between the Huangshan Mountain Range and the Tianmu Mountain Range, with its terrain dominated by medium and low mountains and hills. The region features a humid subtropical monsoon climate, with an annual average temperature ranging from 15.5 to 16.8 ℃, four distinct seasons, and abundant precipitation. Its forest-cover rate ranges from 78% to 85%, and the region with lush vegetation dominated by subtropical evergreen broad‑leaved forests and coniferous‑broad‑leaved mixed forests [19,20].With the rapid growth of spontaneous tourism and the continuous advancement of rural infrastructure construction, the HHAT, as a popular hiking route and tourist destination, is confronting increasing pressure on its surrounding ecological and social environments. Consequently, research on biocultural diversity conservation within this framework is essential for the holistic protection and development of the HHAT area (Fig 2).

**Fig 2:**
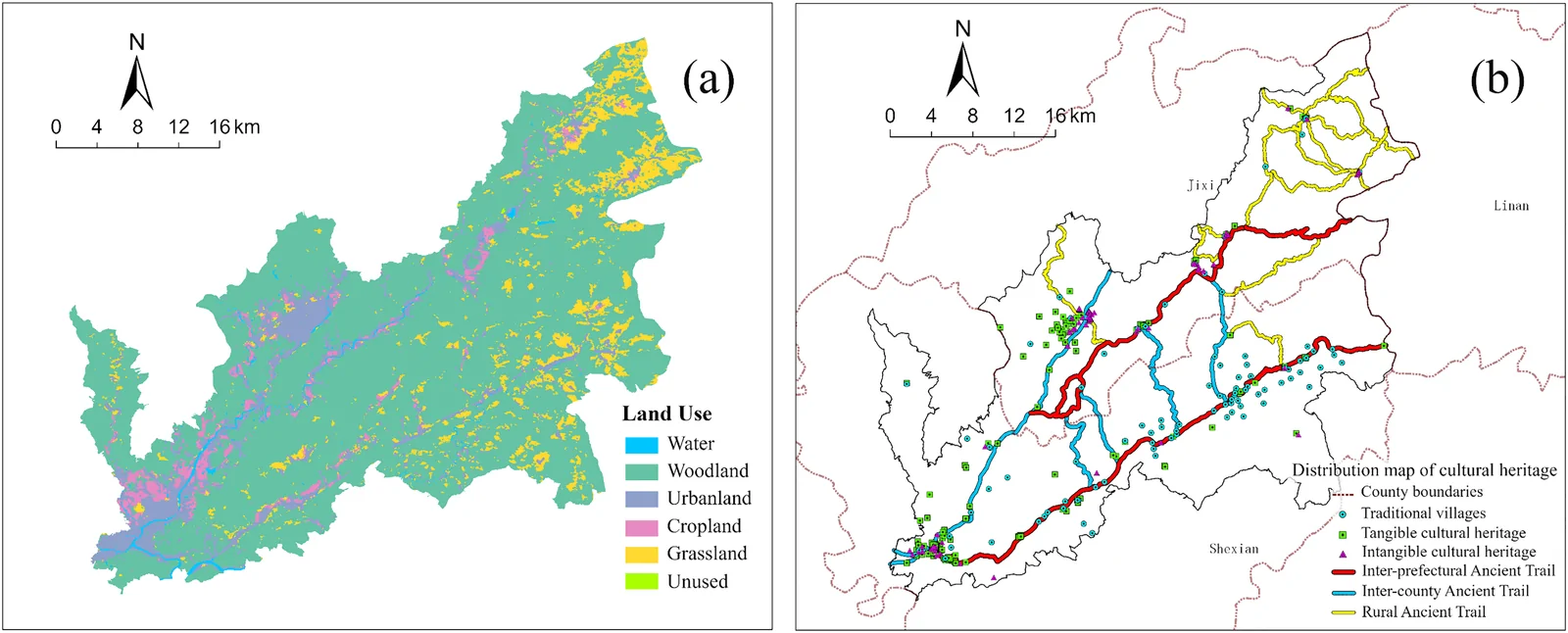
Current status of natural and cultural resources in the HHAT area. (a) Land use distribution map of the study area; (b) Cultural heritage distribution map of the study area. HHAT: Huizhou-Hangzhou Ancient Trail. Note: This figure was created using map data from Tianditu, available at http://www.tianditu.gov.cn/.

### 2.2 Data sources

Data on national and provincial key cultural relics protection units, the national list of traditional villages, and national, provincial, and municipal intangible cultural heritage were all sourced from the culture and tourism bureaus of respective cities and counties. The 2020 land use data were obtained from Esri (https://www.arcgis.com/). Digital Elevation Model (DEM) data were obtained from the Copernicus Global DEM dataset (https://dataspace.copernicus.eu/). Soil type data were obtained from the China Soil Dataset (v1.2) of the World Soil Database. The Normalized Difference Vegetation Index (NDVI) dataset was derived from the MYD13Q1 (Version 6.1) product hosted on the Google Earth Engine (GEE) platform and processed using clipping and raster-calculation functions via the GEE JavaScript API. Precipitation and temperature data were obtained from the National Tibetan Plateau Data Center (https://data.tpdc.ac.cn/). Administrative boundary data were obtained from Tianditu (National Platform for Common Geospatial Information Services; https://www.tianditu.gov.cn/). Road data were obtained from the Earth Resources Data Cloud Platform (http://www.gis5g.com). The 2020 human footprint data was provided by the Urban Environment and Mega-city Monitoring (UEMM) Group, College of Land Science and Technology, China Agricultural University. All these data were clipped using ArcGIS Pro, uniformly projected to the WGS_1984_UTM_zone_50N projection, and resampled to a 30m resolution.

## 3 Methods

### 3.1 Biocultural diversity index

#### 3.1.1 Biodiversity index.

Referring to the research methods of Yang [21] and Zhao [22], this study selects five indicators (Table 2) including Habitat Quality, Net Primary Productivity, Habitat Area Percentage, Normalized Difference Vegetation Index, and Landscape Diversity Index to construct the Biodiversity Index (BD). The formula is as follows:

**Table 2:** Biodiversity assessment indicators.

Indicators	Meaning of the indicators	Formula
Habitat quality index	Habitat quality is a measure of an ecosystem’s capacity to support the survival and reproductive needs of species, reflecting the potential for species to thrive and reproduce in a given habitat [23].	$Q_{ij}=H_{j}\left[\text{1}-\left(\frac{D_{ij}^{z}}{D_{ij}^{z}+K^{z}}\right)\right]$ where *Q_ij_* is the habitat quality of pixel *i* in ecosystem *j*, while *D_ij_* represents the habitat stress level of pixel *i* in ecosystem *j*. The parameter *K* is the half-saturation constant, typically taken as half of the maximum habitat degradation value. The parameter *z* is the normalization constant, typically set to 2.5.
Net primary productivity	According to the *Productivity Hypothesis* based on the *Species-Energy Theory* there is a positive relationship between ecosystem productivity and species richness. Higher levels of ecosystem productivity are associated with greater species abundance [24].	NPP*x* = APAR(*x*,*t*)×*ε*(*x*,*t*) where APAR (*x*, *t*) is the photosynthetically active radiation of grid cell *x* in month *t*, *ε* (*x*, *t*) is the actual light use efficiency of grid cell x in month *t*.
Habitat area percentage	The size of the habitat directly impacts the population density of organisms, with larger habitat patches offering improved conditions for their survival and abundance.	$S_{p}=\frac{A_{e}}{{\text{A}}_{t}}$ where *S_p_* represents the proportion of habitat area; *A_e_* is the habitat area, and *A_t_* is the total area of the ecosystem
Normalized difference vegetation index	NDVI serves as an indicator of photosynthetic activity in vegetation and is also a vegetation index that predicts species richness and diversity [25].	$\text{NDVI}=\frac{\rho_{\text{nir}}-\rho_{\text{red}}}{\rho_{\text{nir}}+\rho_{\text{red}}}$ where *ρ*_nir_ represents the reflectance in the near-infrared band of MODIS, and *ρ*_red_ represents the reflectance in the red band of MODIS
Landscape diversity index	The more balanced the landscape categories, the more complex the ecosystem, and the better the landscape structure, the greater the regional landscape biodiversity [26].	$\begin{array}{c} \text{LDI}=-\sum_{\text{k }=\;\text{1}}^{\text{n}}{\text{P}}_{\text{k}}\text{In}{\text{P}}_{\text{k}},\text{K}=\text{1},\text{2}\cdots \;,\text{n} \end{array}$ where *P*_k_ represents the probability of occurrence of the nth ecosystem type


$$\begin{array}{c} BD=\text{HQI}\times \beta_{\text{1}}+\text{NPP}\times \beta_{\text{2}}+S_{p}\times \beta_{\text{3}}+\text{NDVI}\times \beta_{\text{4}}+\text{LDI}\times \beta_{\text{5}} \end{array}$$ (1)

where *BD* is the Biodiversity Index. *HQI* is Habitat Quality Index. *NPP* is Net Primary Productivity. *S*_*p*_ is Habitat Area Percentage. *NDVI* is the Normalized Difference Vegetation Index. *LDI* is Landscape Diversity Index. *β*_*i*_ represents the weight of each sub-indicator, which was determined by combining the Expert Scoring Method and the Analytic Hierarchy Process (AHP).

Nine experts with rich experience in ecological assessment, whose research fields cover landscape ecology, biodiversity conservation and ecological remote sensing, were invited to independently fill out the pairwise comparison matrices based on the 1–7 scale rule. After geometric mean aggregation of the 9 valid matrices, the eigenvalue method was adopted to calculate indicator weights, followed by consistency tests. The consistency ratio (CR) of each individual expert matrix was less than 0.1, and the CR of the aggregated comprehensive matrix was 0.072, which is below the threshold of 0.1, verifying the validity of the scoring results. The final weights are as follows: *β*_*1*_ = 0.29, *β*_*2* =_ 0.18, *β*_*3*_ = 0.11, *β*_*4*_ = 0.11, *β*_*5*_ = 0.31.

#### 3.1.2 Spatial aggregation degree of cultural heritage.

Kernel density analysis, as a quantitative method commonly used to identify the agglomeration characteristics of spatial point elements, has been widely applied to the research on the spatial patterns of linear cultural heritage (e.g., the Qin-Shu Ancient Road [27], the Eastern Zhejiang Tang Poetry Road [28]). This study adopts this method to characterize the agglomeration patterns and spatial distribution of cultural heritage sites along the HHAT. Cultural heritage encompasses both tangible and intangible categories. A total of 284 tangible cultural heritage sites were compiled in this dataset, covering ancient passes, cliff inscriptions, ancient villages, Huizhou vernacular dwellings and ancestral halls, memorial archways, educational buildings, ancient trail water conservancy facilities, ruins and tombs. The basic vector datasets were extracted and vectorized from cultural relic census inventories released by government authorities at all administrative levels in Anhui Province. GIS software, combined with official online geospatial imagery, was used for spatial georeferencing, verification and correction of heritage locations. The 137 intangible cultural heritage items include Huizhou merchant folk customs, traditional crafts, and folk operas; their core inheriting towns and villages were geocoded within the GIS environment based on provincial intangible cultural heritage inventories.

This study adopts the Rosenblatt-Parzen kernel estimator, and the formula is as follows:


$$\begin{array}{c} \text{g}(x)=\frac{\text{1}}{\text{n}h}\sum_{i=\text{1}}^{n}k\left(\frac{x-X_{i}}{h}\right) \end{array}$$ (2)

where *k* is the kernel function. *h* > 0 is the bandwidth. (*x* − *X*_*i*_) is the distance from the estimation point x to the event *X*_*i*_. Kernel density analysis was implemented via the Spatial Analyst module in ArcGIS Pro. The tool automatically calculates the global bandwidth using a spatially improved algorithm based on Silverman’s rule of thumb, yielding a default bandwidth of 5006.25 m for this dataset. To meet research objectives, three bandwidths (1200 m, 2500 m, and 5006.25 m) were selected for comparative analysis. Bandwidths of 1200 m and 2500 m can reveal localized clustered patches within individual villages yet fail to intuitively reflect the continuous zonal pattern along the HHAT. In contrast, the 5006.25 m bandwidth moderately smooths point features, connects multiple heritage agglomerations along the trail, and clearly identifies the continuous high-density zone formed alongside the ancient trails. This bandwidth better matches the aim of detecting the macroscopic agglomeration framework of the heritage corridor. Accordingly, 5006.25 m was adopted as the analysis bandwidth, with the output cell size set to 30 m. Density values were computed using the built-in Rosenblatt-Parzen quartic kernel function. The Fuzzy Membership tool in ArcGIS Pro was applied to standardize the raw density rasters; raster values were linearly rescaled to the range [0, 1] to eliminate dimensional differences. The standardized layers were subsequently used for weighted overlay analysis of tangible and intangible cultural heritage datasets.

By overlaying the spatial agglomeration degree of material cultural heritage and that of intangible cultural heritage, the spatial distribution map of cultural heritage in the HHAT area is obtained [22]. The formula is as follows:


$$\begin{array}{c} CH=\frac{MCH\;+\;ICH}{\text{2}} \end{array}$$ (3)

where *CH* is the spatial agglomeration degree of cultural heritage, which serves as the proxy indicator for cultural diversity in this study. *MCH* is the spatial agglomeration degree of material cultural heritage. *ICH* is the spatial agglomeration degree of intangible cultural heritage.

Considering that tangible and intangible cultural heritage exert equally important effects on the persistence of the linear cultural corridor and the representation of cultural diversity, and neither category is subordinate to the other, this study follows the findings of Zhao et al. [22]. The standardized agglomeration layers of the two heritage types (both normalized to the range of [0,1]) were overlaid with equal weights to calculate the cultural heritage agglomeration index using an equal-weighted average, producing a comprehensive spatial distribution map of cultural heritage agglomeration across the entire HHAT.

#### 3.1.3 Biocultural diversity index.

The Biocultural Diversity Index (BCD) is calculated using the equal-weighted average method [29]. The formula is as follows:


$$\begin{array}{c} BCD=\frac{BD\;+\;CH}{\text{2}} \end{array}$$ (4)

where *BCD* is the Biocultural Diversity Index. *BD* is the Biodiversity Index. *CH* is the spatial agglomeration degree of cultural heritage.

### 3.2 Calculation of coupling‑coordination degree

#### 3.2.1 Coupling degree model.

The coupling coordination degree is used to analyze the coupling relationships among systems [30]. The formula is as follows:


$$\begin{array}{c} C={\left[\frac{\prod_{i=\text{1}}^{n}U_{i}}{{\left(\frac{\text{1}}{n}\sum_{i=\text{1}}^{n}U_{i}\right)}^{n}}\right]}^{\frac{\text{1}}{n}}=\sqrt{\frac{U_{\text{1}}U_{\text{2}}}{{\left(\frac{U_{\text{1}}+U_{\text{2}}}{\text{2}}\right)}^{\text{2}}}}=\frac{\text{2}\sqrt{U_{\text{1}}U_{\text{2}}}}{U_{\text{1}}+U_{\text{2}}} \end{array}$$ (5)

$$\begin{array}{c} T=\sum_{i=\text{1}}^{n}\alpha_{i}\times U_{i,}\sum_{i=\text{1}}^{n}\alpha_{i}=\text{1} \end{array}$$ (6)

$$\begin{array}{c} D=\sqrt{C\times T} \end{array}$$ (7)

where *C* is the coupling degree with a value range of [0,1]. T is the comprehensive coordination index of the two systems. *D* is the coupling coordination degree with a value range of [0,1]. *n* is the number of systems. *U*_*1*_ and *U*_*2*_ are represented by the raster data of cultural diversity and biodiversity results. *U*_*1*_ and *U*_*2*_ were normalized to the interval of [0,1] using the Fuzzy Membership tool in ArcGIS Pro. *αᵢ* is the weight of the *i*-th system. In this study, biodiversity and cultural diversity are regarded as two systems of equal importance, so *αᵢ* is assigned a value of 0.5.

#### 3.2.2 Relative development degree model.

The relative development degree can reveal the relative development levels of biodiversity and cultural diversity. Therefore, this study introduces the relative development degree model to calculate the relative development coefficient between biodiversity and cultural diversity.


$$\begin{array}{c} \gamma =\frac{U_{\text{1}}}{U_{\text{2}}} \end{array}$$ (8)

where γ represents the relative development degree, *U*_*1*_ denotes the cultural diversity index, and *U*_*2*_ denotes the biodiversity index. Referring to previous studies [31–33], this study classifies the coupling‑coordination development states and types of biodiversity and cultural diversity (Table 3).

**Table 3:** Phases and types of coupling coordination development between biodiversity index and cultural diversity.

Coupling coordination degree	Relative development degree	Type	Characteristics of coupling coordination development	Development stage
0 < D ≤ 0.5	0 < γ ≤ 0.8	Ⅰ	Cultural diversity lags behind biodiversity, with high antagonism between the two.	Antagonism
	0.8 < γ ≤ 1.2	Ⅱ	Cultural diversity is synchronized with biodiversity, with low antagonism between the two.	
	γ > 1.2	Ⅲ	Cultural diversity precedes biodiversity, with high antagonism between the two.	
0.5 < D ≤ 0.7	0 < γ ≤ 0.8	Ⅳ	Cultural diversity lags behind biodiversity, with low running‑in between the two.	Running‑in
	0.8 < γ ≤ 1.2	Ⅴ	Cultural diversity is synchronized with biodiversity, with high running‑in between the two.	
	γ > 1.2	Ⅵ	Cultural diversity precedes biodiversity, with low running‑in between the two.	
0.7 < D ≤ 1	0 < γ ≤ 0.8	Ⅶ	Cultural diversity lags behind biodiversity, with low coordination between the two.	Coordination
	0.8 < γ ≤ 1.2	Ⅷ	Cultural diversity is synchronized with biodiversity, with high coordination between the two.	
	γ > 1.2	Ⅸ	Cultural diversity precedes biodiversity, with low coordination between the two.	

### 3.3 Priority conservation area identification based on the Marxan model

The Marxan model was originally developed by Ian Ball and Hugh Possingham at the University of Queensland. As a multi-objective systematic conservation planning software based on the simulated annealing algorithm, it can achieve the most effective conservation goals under certain conservation cost constraints [34,35]. The basic objective function of the Marxan model is as follows:


$$\begin{array}{c} Sc=\sum Cost+\sum SPF\times Penalty+BLM\sum Boundary \end{array}$$ (9)

where *Sc* is the total conservation cost. *Cost* is the conservation cost of a single grid. *SPF* is the penalty for a single grid failing to meet conservation targets. *Penalty* is the penalty coefficient. *BLM* is a weighting parameter to balance conservation cost and total boundary length of selected planning units. A larger *BLM* assigns greater weight to the boundary term, and the model tends to produce more compact conservation patches with shorter total boundary length.

This study adopted 300 m × 300 m homogeneous grids as Marxan planning units. Uneven township sizes and artificial administrative boundaries fragment linear corridors and cause the modifiable areal unit problem, interfering with conservation pattern identification. We compared 300 m, 500 m and 1000 m grid schemes: the 1000 m grid blurs heritage hotspots, and the 500 m grid cannot distinguish adjacent heritage clusters. Finally, 14,760 300 m grid units were generated to capture fine biocultural spatial differentiation. The 2020 human footprint index was used as the cost proxy. With reference to existing studies, 8 evaluation factors for biocultural diversity were identified as conservation targets [36,37]. Specifically, for the areal data of biodiversity, the high-value areas accounting for 15%–30% of the total area within their respective planning units were selected; for the point data of cultural diversity, all such data were fully included. Based on the 30% conservation target proposed by the International Union for Conservation of Nature (IUCN) and the research of Zhang [38] and Zhao [39], combined with the sensitivity test of biodiversity conservation targets, the threshold of biodiversity conservation targets was set at 30%, and that of cultural diversity conservation targets at 60%.

In terms of parameter settings, multiple pre-experiments showed that the conservation targets could all be met when the species penalty factor (SPF) value was set to 1 for all conservation targets; thus, the SPF value was determined as 1. This study set the BLM values into six levels (0, 0.01, 1, 10, 100, 1000) and performed 100 runs. A curve was then plotted to show the variation of conservation costs with boundary lengths under different BLM values. The results indicated that the inflection point value of 2.06 was the optimal BLM value. Finally, with the number of iterations set to 100 in Marxan, priority protected areas were delineated according to the selection frequency of each planning unit.

### 3.4 Classification of conservation types

To further determine the conservation strategies for the HHAT area, overlay analysis was first conducted on the BCD, the results generated by the Marxan model, and the protected areas along the ancient trails. The integrated results were then output as the core protected areas of the study region. Subsequently, the core protected areas were combined with the current status of the ancient trails to classify four distinct conservation types for the trails, with the specific operation process illustrated in Fig 3.

**Fig 3:**
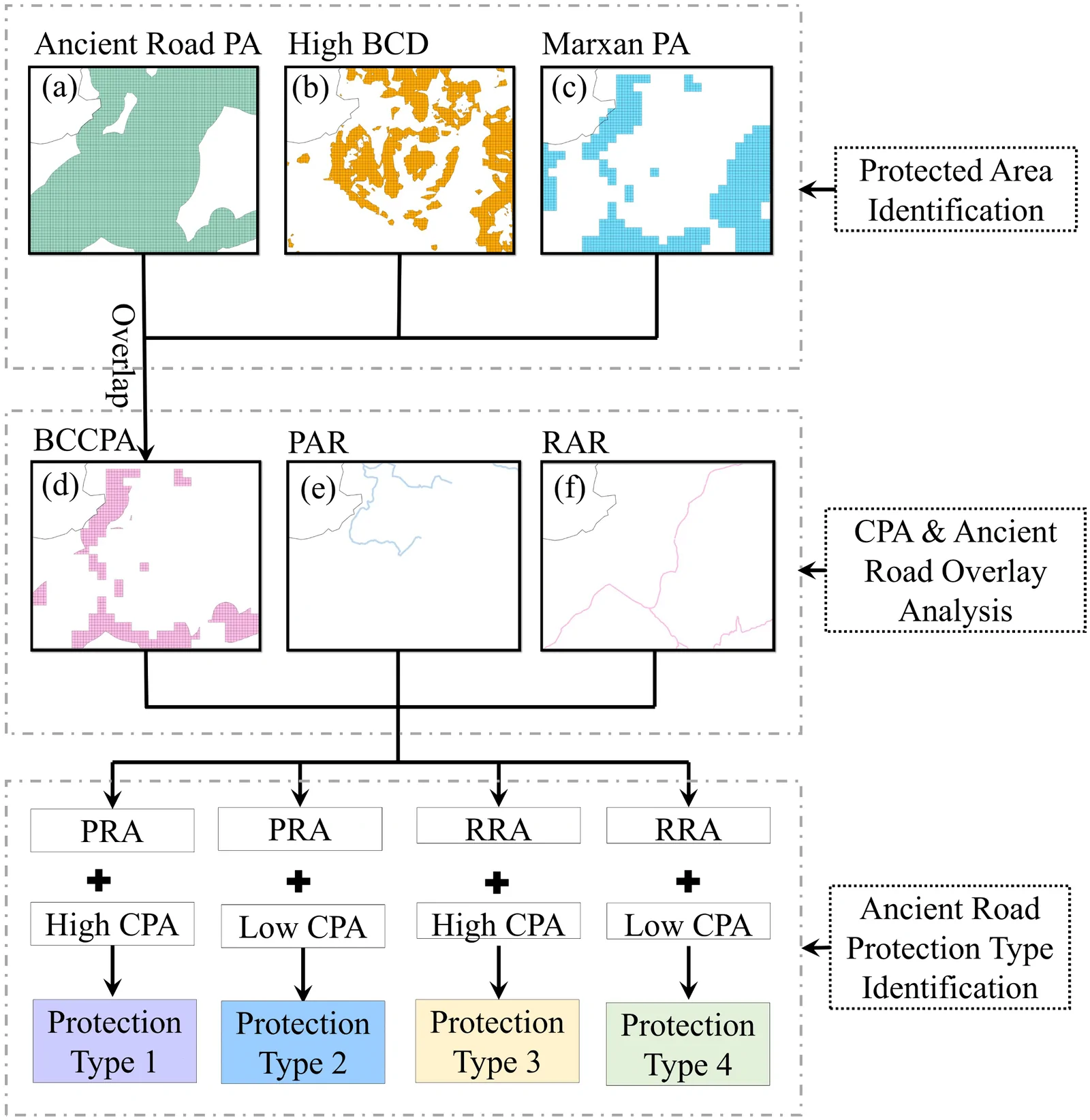
Process diagram for the classification of conservation types. (a) Protected areas along the ancient trails; (b) High-value zones of biocultural assessment; (c) Protected areas identified by the Marxan model; (d) Core protected areas; (e) Preserved sections of the ancient trails; (f) Reconstructed sections of the ancient trails.

## 4 Results

### 4.1 Spatial patterns of biocultural diversity along the HHAT

Figs 4 and 5 present the assessment results of biodiversity and cultural diversity, respectively. As shown in Fig 4f and Fig 5d, both biodiversity and cultural diversity along the HHAT exhibit pronounced spatial heterogeneity. Areas with high biodiversity values are predominantly concentrated in mountainous regions, generally at elevations above 400 m (Fig 4f), with particularly high levels observed in areas exceeding 700 m. These regions are mainly covered by forests and are often distributed around mountainous villages and forestry farms, where ecological conditions are well preserved, whereas areas with low biodiversity values are primarily associated with urban construction land and are largely concentrated in low-altitude areas.

**Fig 4:**
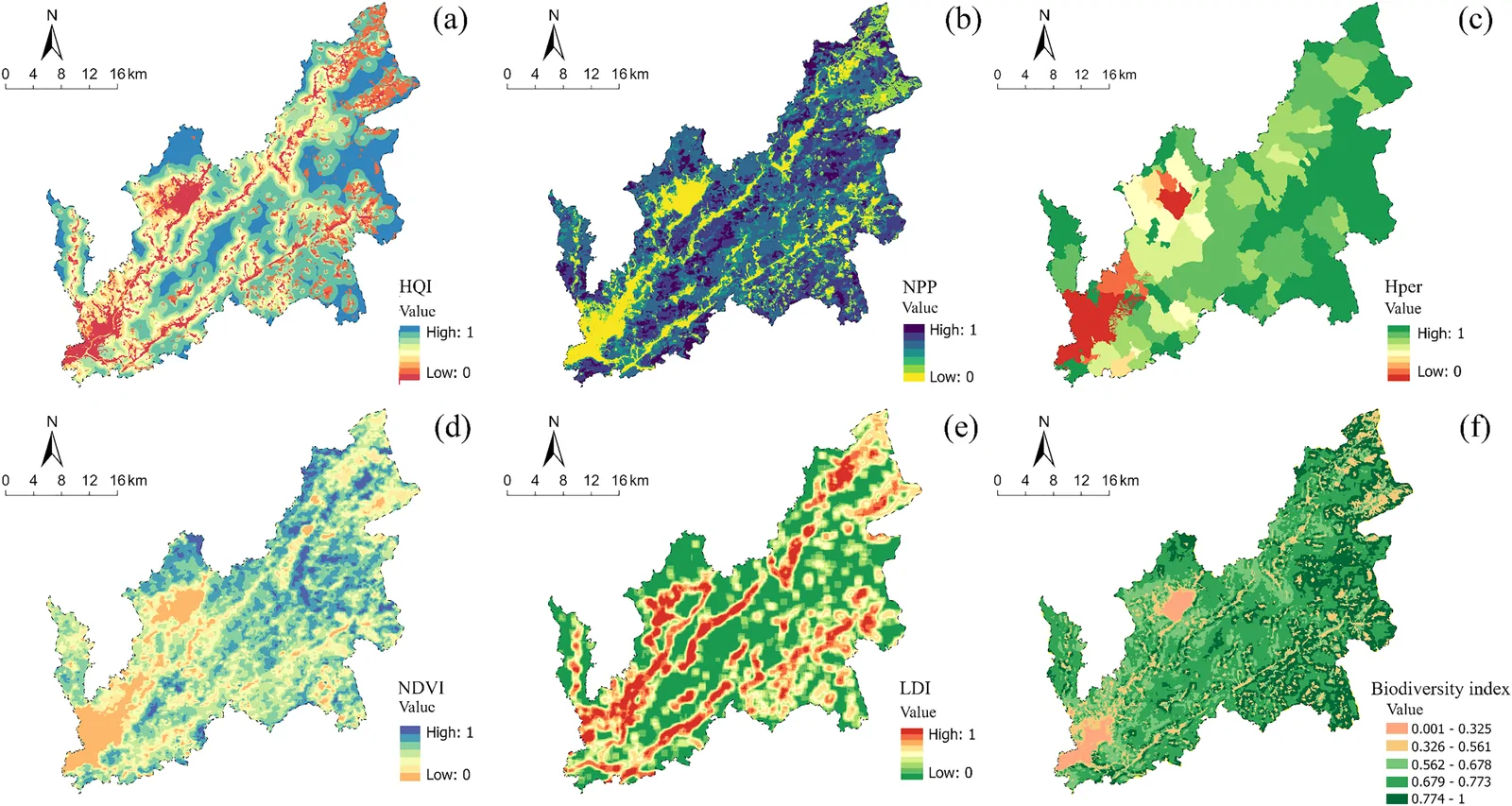
Results of the biodiversity assessment. (a) Habitat Quality Index (HQI); (b) Normalized Difference Vegetation Index (NDVI); (c) Net Primary Productivity Index (NPP); (d) Landscape Diversity Index (LDI); (e) Habitat Area Percentage (Hper); (f) Biodiversity Index (BD). Note: This figure was created using map data from Tianditu, available at http://www.tianditu.gov.cn/.

**Fig 5:**
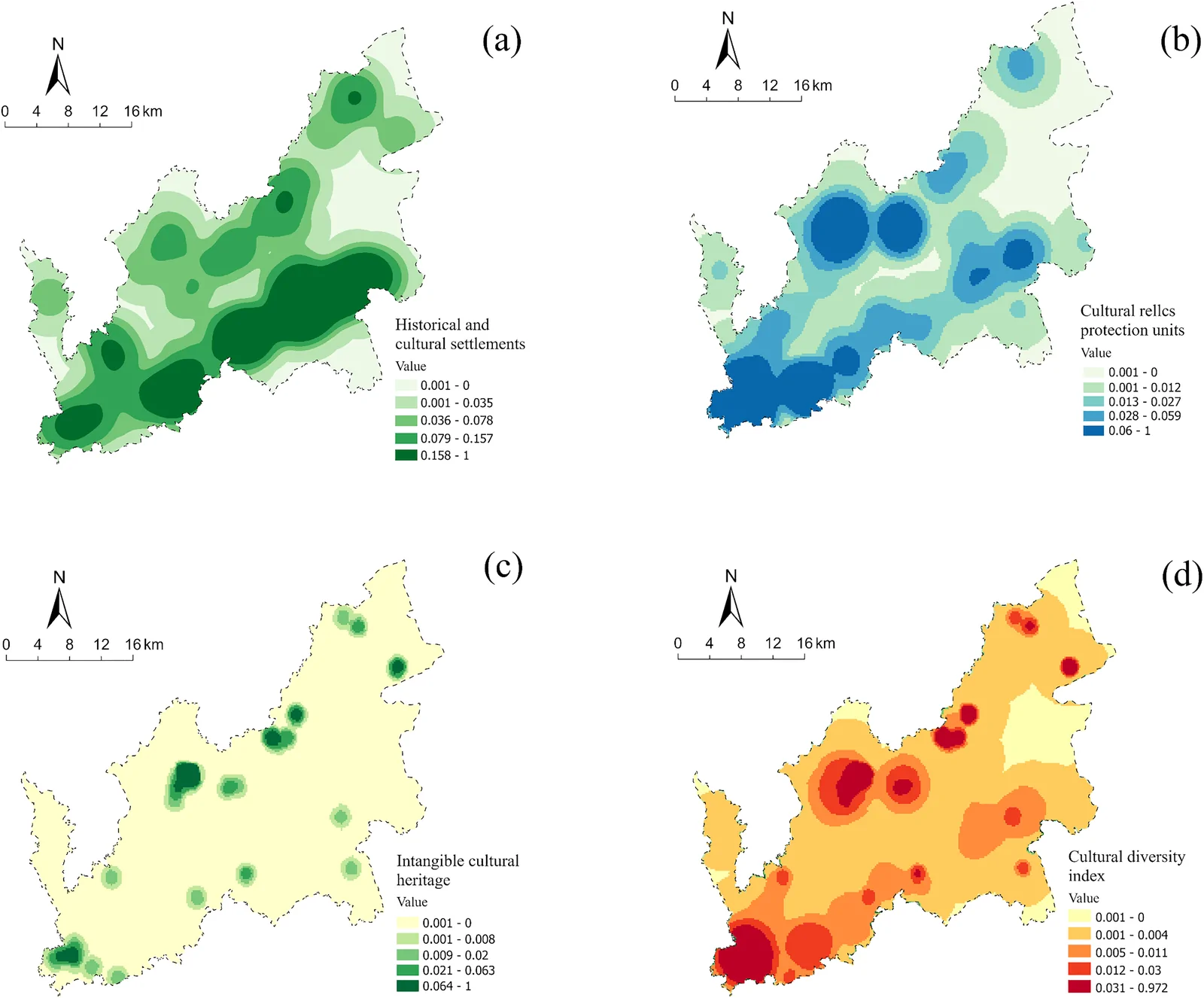
Results of the cultural diversity assessment. (a) Spatial aggregation of traditional villages; (b) Spatial aggregation of cultural heritage sites; (c) Spatial concentration of intangible cultural heritage; (d) Cultural diversity index. Note: This figure was created using map data from Tianditu, available at http://www.tianditu.gov.cn/.

In contrast, areas with high cultural diversity values (Fig 5d) are mainly concentrated around the major urban centers of Jixi County and She County. These two regions served as economic and cultural hubs during the ancient Huizhou period, leaving behind abundant tangible and intangible cultural heritage. Other high-value areas are primarily distributed along the Dengyuan AT and the Huichang AT, as well as in villages and towns along these routes, such as Yingzhou Town and Hucun Village in Fuling Town of Jixi County, and Zhanqi Village in Bei’an Town, Xiakeng Village in Xiakeng Town, and Sanyang Village in Sanyang Township of She County. These areas are geographically close to the ancient trails and are characterized by well-developed transportation networks, low and relatively flat terrain, concentrated settlements, and rich material and intangible cultural heritage.

By overlaying the biodiversity and cultural heritage diversity results, a biocultural diversity index was derived and classified into three levels using the natural breaks method: low biocultural diversity (0.015–0.288), medium biocultural diversity (0.289–0.370), and high biocultural diversity (0.371–0.650) (Fig 6). The results indicate that areas with low biocultural diversity are mainly concentrated in urban built-up land and high-altitude grasslands, whereas high-value areas are predominantly located in high-altitude regions and are scattered around urban peripheries as well as along major ancient trail corridors.

**Fig 6:**
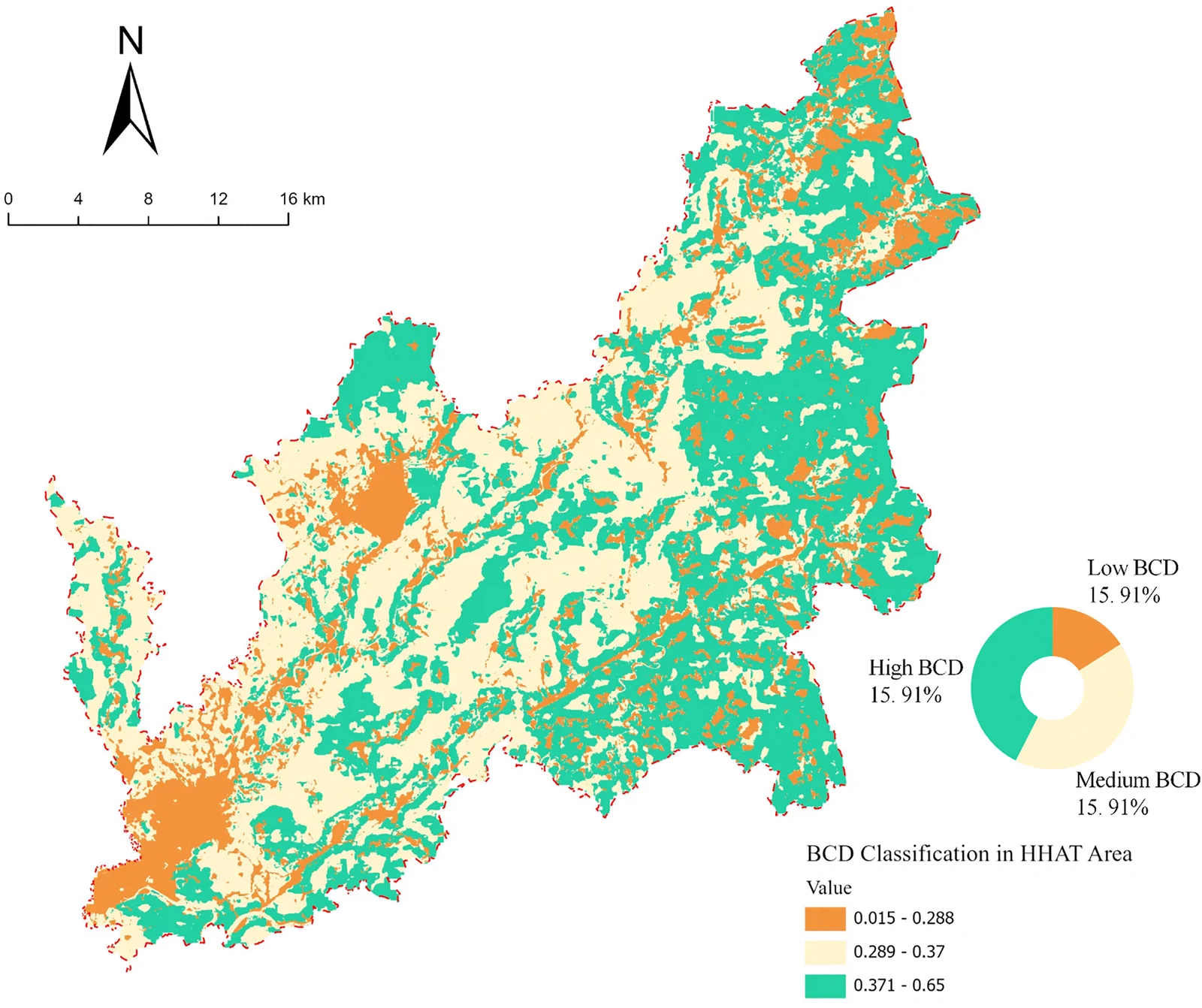
BCD Classification in the HHAT area. BCD: Biocultural Diversity Index; HHAT: Huizhou-Hangzhou Ancient Trail. Note: This figure was created using map data from Tianditu, available at http://www.tianditu.gov.cn/.

From a holistic perspective, only a few areas exhibit both high levels of biodiversity and cultural diversity. Most areas, however, display pronounced spatial disjunction, manifested as relatively scarce cultural elements in regions with high biodiversity, or low biodiversity in areas rich in cultural heritage. This spatial differentiation provides an empirical basis for identifying biocultural synergy zones and potential trade-off areas.

### 4.2 Coupling and coordination characteristics of biocultural diversity along the HHAT

The results of coupling coordination degree and coordinated development degree between the biodiversity index and cultural diversity index are illustrated in Fig 7, and the statistical outcomes are presented in Table 4. The results reveal that the antagonistic state dominates the coupling‑coordination relationships across the study area, accounting for 98.90%, and most of these areas belong to type Ⅰ, where cultural diversity lags behind biodiversity with prominent antagonistic effects between the two dimensions. The running‑in state accounts for 1.08%, mainly distributed in the main urban areas of Jixi County and She County, as well as villages along the ancient trail including Shanghujia, Fuling and Hucun. The coordinated state only occupies 0.02%, located primarily at the junction east of Jixi County urban area and an adjacent forest farm.

**Fig 7:**
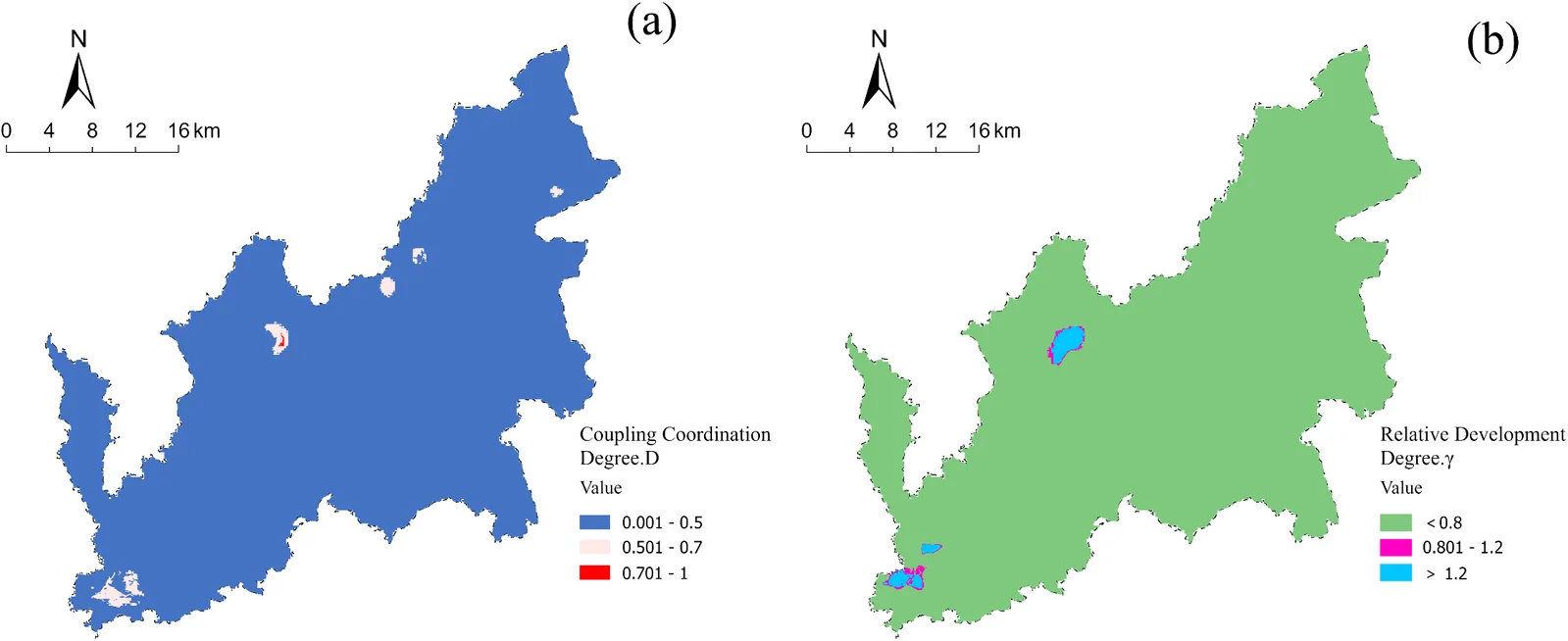
Results of coupling‑coordination and relative development analyses. (a) Biodiversity and cultural diversity coupling coordination degree; (b) Biodiversity and cultural diversity relative development degree. Note: This figure was created using map data from Tianditu, available at http://www.tianditu.gov.cn/.

**Table 4:** Calculation of coupling‑coordinated development for biodiversity and cultural diversity.

Coupling coordination degree	Relative development degree	Type	Area(km^2^)	Proportion (%)	Development stage
0 < D ≤ 0.5	0 < γ ≤ 0.8	Ⅰ	1227.27	97.940	Antagonism
	0.8 < γ ≤ 1.2	Ⅱ	3.12	0.249	
	γ > 1.2	Ⅲ	8.89	0.709	
0.5 < D ≤ 0.7	0 < γ ≤ 0.8	Ⅳ	11.33	0.904	Running‑in
	0.8 < γ ≤ 1.2	Ⅴ	0.63	0.050	
	γ > 1.2	Ⅵ	1.60	0.128	
0.7 < D ≤ 1	0 < γ ≤ 0.8	Ⅶ	0.91	0.007	Coordination
	0.8 < γ ≤ 1.2	Ⅷ	0.95	0.008	
	γ > 1.2	Ⅸ	0.45	0.004	

These results demonstrates that the entire HHAT exhibits a pronounced antagonistic coupling relationship. Regions with high biodiversity values tend to have low cultural diversity, whereas areas with abundant cultural resources and dense traditional settlements show relatively insufficient biodiversity. The overall low values of coupling coordination degree indicate poor synchronicity in the variation of the two indices, suggesting a potential spatial trade‑off between biodiversity and cultural diversity. Overall, the level of biocultural coordinated development along the HHAT remains generally weak, which may correspond to the spatial pattern of divergence between natural ecological conditions and human cultural activities.

### 4.3 Results of the conservation type zoning for the HHAT

#### 4.3.1 Optimal protected area identification results.

Based on the selection frequency of protected units derived from 100 iterations of the Marxan model simulation (Fig 8), this study divided the research area into six tiers of protected zones (Fig 8a). Furthermore, planning units with selection frequencies between 80 and 100 iterations were designated as the optimal protection zone for the HHAT (Fig 8b). As shown in Fig 8b, the optimal protection zone for the HHAT covers approximately 438.57 km^2^. Spatially, this zone exhibits significant aggregation, forming large contiguous clusters in the central and eastern parts of the study area, while a large concentrated patch also exists in the west. Overall, a core-periphery spatial pattern emerges: the core area exhibits high contiguity, while fragmented small patches are scattered in the periphery, highlighting the uneven spatial distribution of protected zones. The contiguous areas predominantly occupy higher-elevation zones with superior ecological foundations, while the scattered patches correspond to villages and townships such as northern Guilin Town, northern Huayang Town, and central Qizili Town. These areas not only feature favorable ecological conditions but also harbor relatively abundant cultural heritage resources.

**Fig 8:**
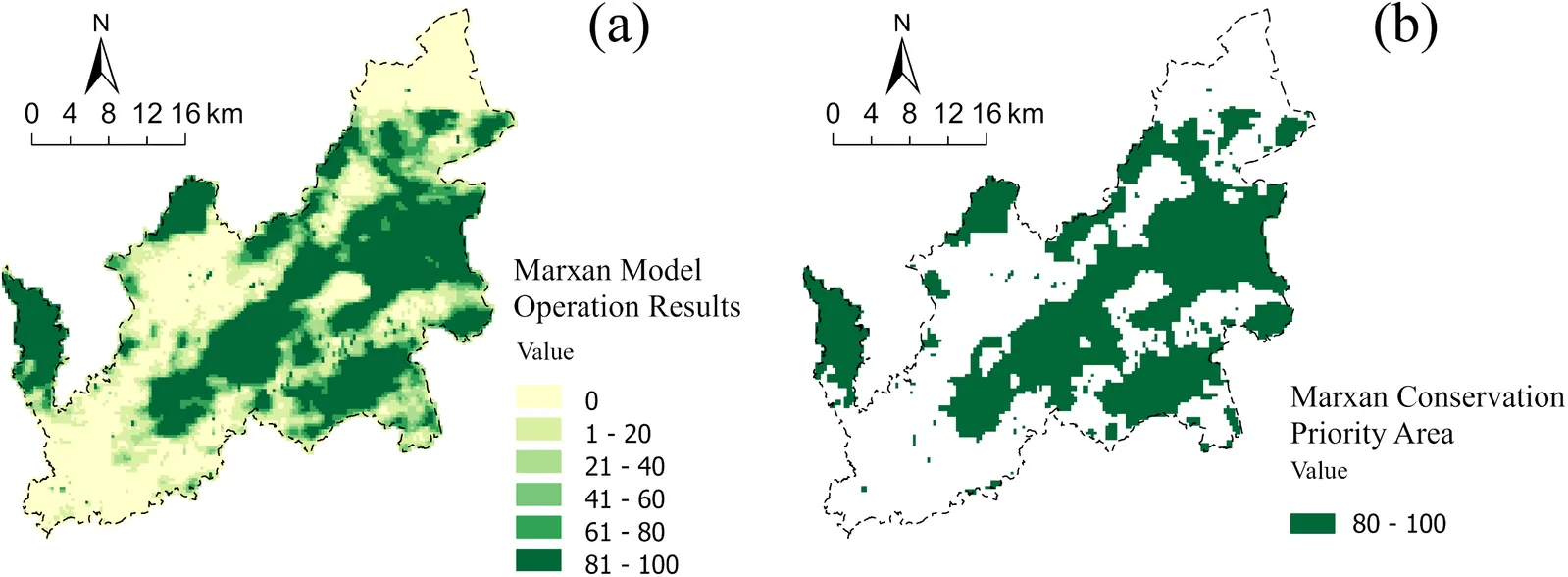
Marxan simulation results. (a) Protection tiers of the study area by Marxan selection frequency; (b)Optimal conservation zone of HHAT. Note: This figure was created using map data from Tianditu, available at http://www.tianditu.gov.cn/.

#### 4.3.2 Types of conservation for the HHAT.

Based on research by Zhang et al. [40], the protection scope for the HHAT was determined by overlaying buffer zones with radii of 1500 m for inter-prefectural ancient trails (covering 90% of heritage sites along the route), 1000 m for inter-county ancient trails (covering 80% of heritage sites along the route), and 500 m for rural ancient trails (covering 70% of heritage sites along the route). Overlaying these with analyses of high-value biocultural diversity zones and optimal conservation areas yields the Biocultural Core Protected Areas (BCCPA) for the HHAT area (Fig 9). Comparison with land use maps reveals that the BCCPA primarily occupies forested areas adjacent to two prefectural-level ancient trails, situated not far from urban peripheries. Fig 9 also shows that the ancient trails within the BCCPA include two prefectural-level trails: the Xiaoyaoling AT and the Laozhuling AT; three county-level trails: the Huanlianling AT, the Wangkengling AT, and the Bancha AT; and six rural trails: the Dazhang AT, the Huiling AT, the Dade AT, the Zhuling AT, the Fenggaoling AT, and the Jingkanling AT. Among these, the Xiaoyaoling AT, Laozhuling AT, Huanlianling AT, Wangkengling AT, Fenggaoling AT, Zhuling AT, Jingkanling AT, Huiling AT, and Dade AT are all preserved ancient trails.

**Fig 9:**
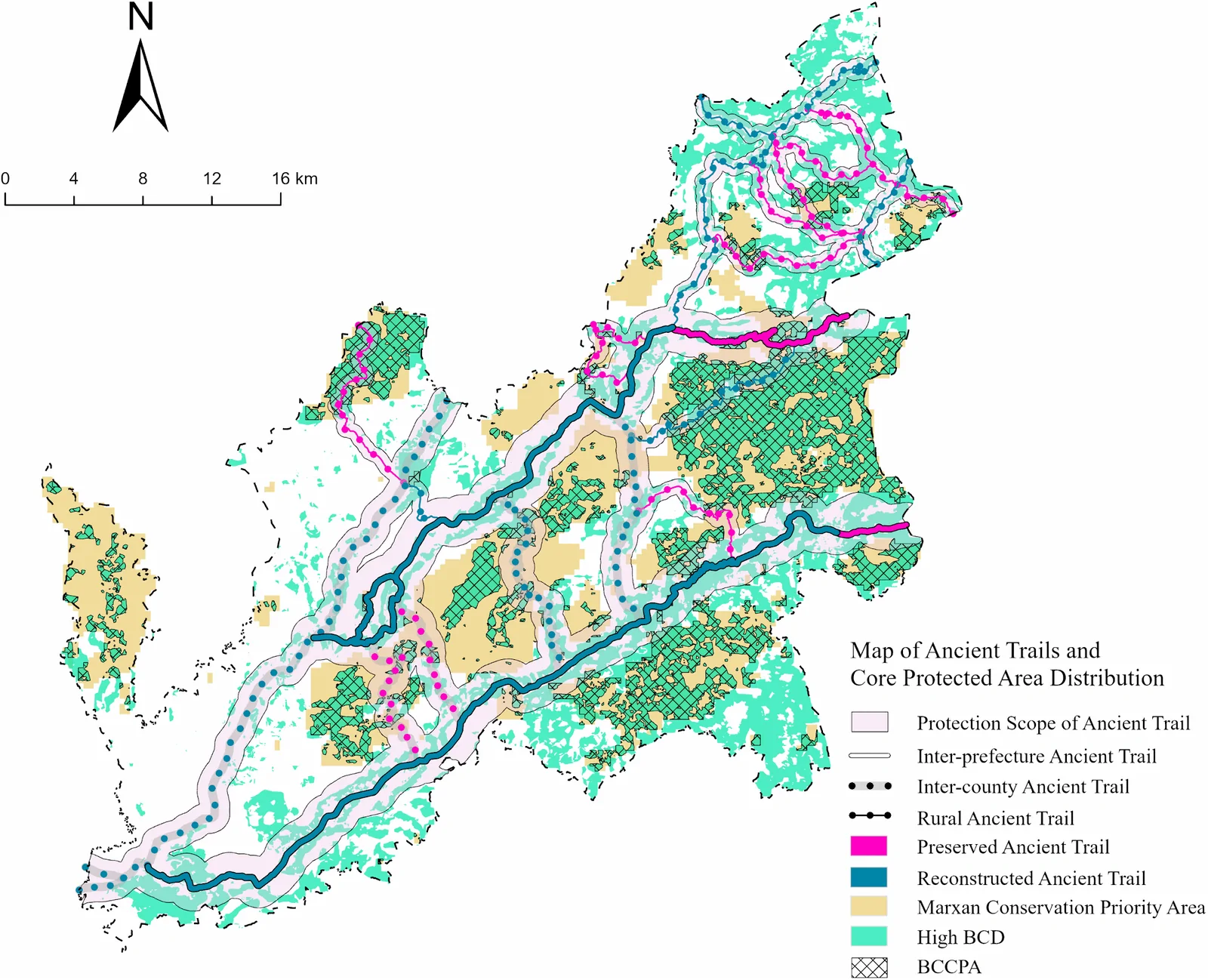
Distribution map of ancient trails and core protected areas.

By calculating the proportion of BCCPA within ancient trails conservation areas, significant variations were observed across different protected zones. The Dazhang AT conservation area contained 52.31% BCCPA, whereas areas like Songyantang AT and Gexiyuan AT contained 0% BCCPA. A total of 26 ancient trails were investigated in this study. Three classification thresholds (5%, 10%, 15%) were tested comparatively: under the 5% threshold, 14 trails were categorized as conservation-oriented, failing to distinguish corridors with vastly different resource endowments; the 15% threshold yielded only four conservation-oriented trails, resulting in an insufficient sample size that obscured the gradient differentiation of corridor resources. The 10% threshold screened out 8 trails with ample core conservation space, clearly separating high-value conservation segments from resource-deficient restoration segments, and was thus adopted as the classification criterion. Based on the current state of the trails, those within protected areas with BCCPA coverage exceeding 10% were classified as conservation-type trails, while those below 10% were designated as restoration-type trails. Consequently, the trails were categorized into four conservation types: Conservation-Minimal Intervention, Conservation-Appropriate Revitalization, Restoration-Minimal Intervention, and Restoration-Appropriate Revitalization (Fig 10).

**Fig 10:**
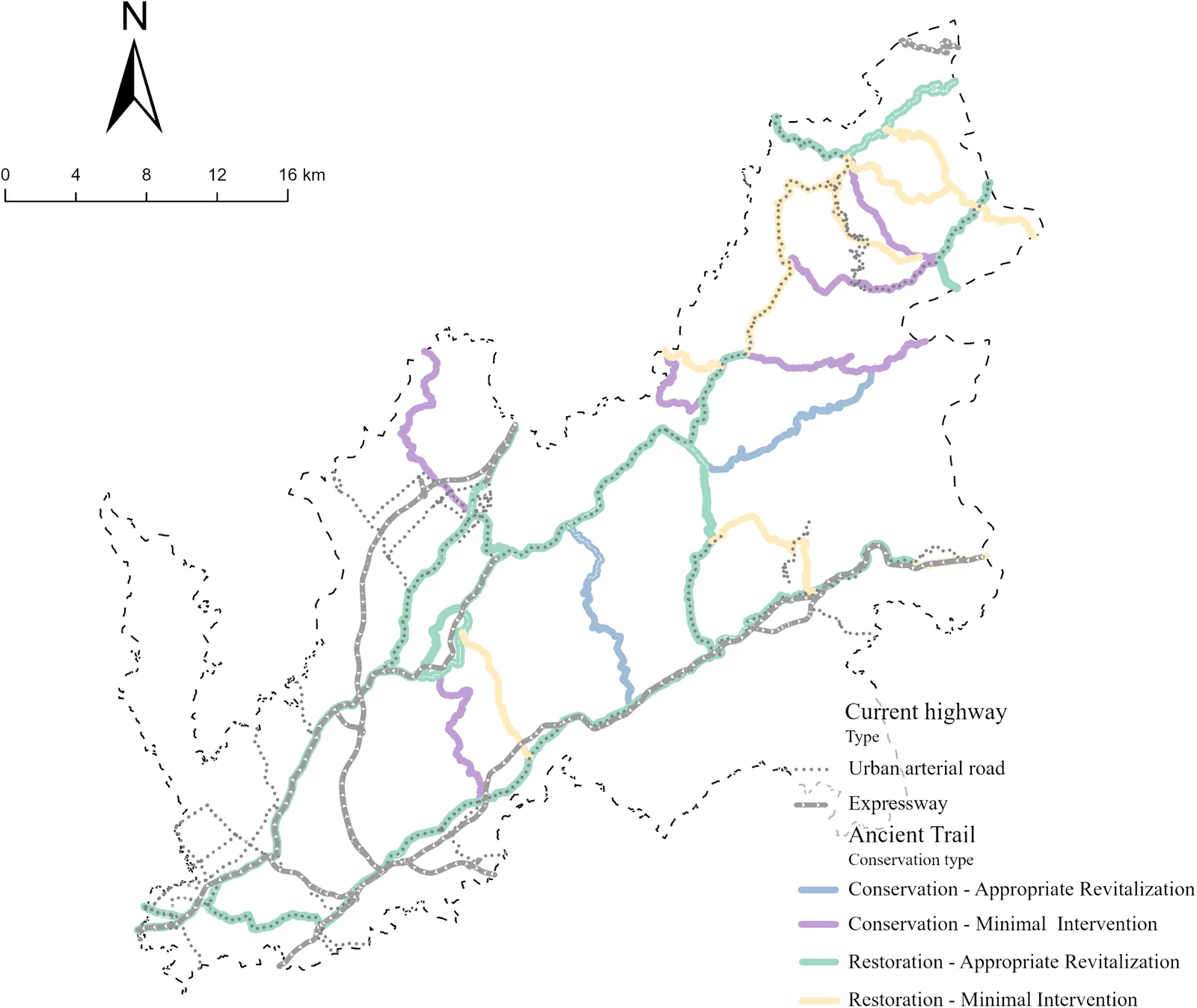
Classification of conservation types for the HHAT. HHAT: Huizhou-Hangzhou Ancient Trail. Note: This figure was created using map data from Tianditu, available at http://www.tianditu.gov.cn/.

Conservation-type ancient trails prioritize protection with minimal intervention, while restoration-type ancient trails may undergo moderate revitalization. Conservation-Minimal Intervention: High BCCPA coverage around the trail, and the trail remains well-preserved. Conservation-Appropriate Revitalization: High BCCPA coverage around the trail, but the trail has been converted into a modern highway. Restoration-Minimal Intervention: Low BCCPA coverage around the trail, but the trail remains well-preserved. Restoration-Appropriate Revitalization: Low BCCPA coverage around the trail, and the trail has been converted into a modern highway.

As shown in Fig 10, the Restoration-Appropriate Revitalization type ancient trails are primarily distributed in low-altitude urban construction land, serving as vital transportation corridors connecting county seats with townships. Typical examples include the Dengyuan AT, Huichang AT, and Congshanguan AT. Meanwhile, the Conservation-Minimal Intervention type, Conservation-Appropriate revitalization type, and Restoration-Minimal Intervention type ancient trails are concentrated in high-altitude forested and grassland areas. Field investigations reveal that high-altitude grasslands within the study area are predominantly cultivated as vegetable plots and cash-crop fields (e.g., chrysanthemums, tea) by local residents. These ancient trails typically serve as connecting pathways between villages and towns, traversing high-altitude mountainous regions. Due to their irreplaceability by modern transportation networks, they maintain robust ecological environments and exhibit high levels of structural preservation.

## 5 Discussion

### 5.1 Biological and cultural diversity: potential explanations for the antagonistic coupling pattern

The core finding of this study is that biodiversity and cultural diversity exhibit a pronounced coupling relationship in the HHAT area. Specifically, areas of high biodiversity are primarily distributed in mid-to-high mountain zones above 400m elevation, characterized by continuous woodlands and natural habitats, while their cultural diversity levels are generally low. Conversely, areas with high cultural diversity are concentrated in low-elevation river valleys and relatively flat terrain, where biodiversity is significantly degraded. Coupling‑coordination analysis further reveals that regions with antagonistic coupling account for 98.90% of the total study area, whereas coordinated regions make up only 0.02%. This observed spatial pattern may suggest a non-synergistic association between biological and cultural systems across the landscape. This characteristic starkly contrasts with the positive correlations or neutral relationships commonly observed in large-scale regions (e.g., the national scale of Colombia [41], the Taihu Basin in China’s Yangtze River Delta [42]), highlighting the uniqueness of linear cultural heritage in small-scale mountainous regions.

Based on the calculation results of the coupling coordination degree, combined with the physical‑geographical and human‑social background of the study area, it is inferred that the formation of this antagonistic coupling pattern may be related to the following three factors. First, rigid competition for spatial resources at a small scale plays a key role. The study area covers only approximately 1,268.94 km^2^, and spaces suitable for human settlement (such as river terraces and gentle slopes) largely overlap with biological habitats. Long-term human activities—including housing construction, agricultural expansion, and road development—have led to vegetation fragmentation, resulting in a pattern of cultural enrichment accompanied by biodiversity decline, whereas remote mountainous areas with limited human disturbance exhibit the opposite pattern of biodiversity enrichment but weak cultural presence [43]. Second, as a historical commercial corridor, the HHAT has promoted the spatial concentration of cultural heritage. However, tourist flows and infrastructure development along the trail have also exerted pressure on local ecosystems. In contrast, remote mountainous areas outside the corridor’s influence suffer from limited accessibility, resulting in weak carriers for cultural heritage transmission [44]. Finally, traditional livelihood practices in the mountainous areas of southern Anhui—such as agricultural reclamation and forest resource utilization—together with cultural inheritance activities (e.g., expansion of ancient villages and folk customs) have long relied on natural space. At small spatial scales, the cumulative effects of human activities further amplify conflicts between ecological systems and cultural development [45].

It is noteworthy that although the spatial covariation of biodiversity and cultural diversity across the HHAT region is dominated by antagonistic coupling, these two components exhibit implicit synergies and dynamic trade-offs over long-term evolution, with important implications for regional conservation and sustainable development [6]. Synergistic interactions operate bidirectionally. Intact mountain ecosystems and biological resources underpin the continuity of Huizhou’s traditional agrarian, folk and ancient trail commercial culture; stable biodiversity sustains resource supplies for traditional livelihoods and cultural heritage transmission [46]. In turn, indigenous ecological perceptions, farming conventions and conservation practices of roadside settlements foster ecological stewardship, mitigating landscape degradation and maintaining mountain ecosystem integrity [47].

Unlike static spatial antagonism, trade-offs represent dynamic decision-making under resource constraints. Endowed with a terrain pattern of “seven parts mountains, one part water and one part farmland”, Huizhou has experienced persistent mountain resource scarcity. Historical and contemporary governance measures—from Ming–Qing clan woodland regulations and reclamation bans to modern tea plantation management and watershed compensation schemes—avoid extreme development or rigid exclusion. Instead, balanced strategies have been adopted: moderate valley development is permitted to sustain livelihoods and cultural inheritance, while steep slopes, ancient trail native vegetation and key water source zones are protected via mandatory conservation red lines. Multi-layered policies confirm enduring trade-offs between ecological conservation and agricultural-cultural development in Huizhou’s mountainous terrain [48–51].

The coexistence of antagonistic, synergistic and trade-off relationships shapes the unique spatial differentiation of dual diversity within this small-scale mountain linear heritage zone. Elucidating such interactions helps reconcile multiple conservation targets, preventing biased overemphasis on either ecological protection or cultural exploitation, and provides integrated theoretical support and practical guidance for coordinated biocultural diversity conservation in mountain linear heritage sites.

### 5.2 Integrated conservation framework and spatial response strategies for the HHAT

In response to the dilemma of antagonistic coupling between biodiversity and cultural diversity, this study proposes an innovative, targeted integrated conservation framework based on the BCCPA identification results and an assessment of the current conditions of the ancient trails. In contrast to conventional segregated planning that handles ecological and cultural elements independently, this framework’s core innovation is the construction of a closed-loop conservation route for quantitatively coupled biocultural dimensions. The combined application of the BCD index and Marxan model enables simultaneous valuation of biological and cultural resources. Together with differentiated hierarchical zoning strategies, this approach mitigates prominent drawbacks of traditional planning: dimensional imbalance and the decoupling of theory from on-the-ground practice. Specifically, the BCD index addresses the imbalance in traditional studies that prioritize either ecological or cultural dimensions, enabling precise identification of core areas with dual ecological and cultural significance [52]. The Marxan model, grounded in systematic conservation planning principles, efficiently identifies optimal priority conservation areas [53]. By coupling these two approaches to delineate core conservation zones and further classifying them into four conservation types in accordance with the actual conditions of the ancient trails, this study ultimately establishes a closed-loop management system of assessment–zoning–implementation. This framework not only provides a robust academic foundation for conservation practice but also facilitates the translation of planning recommendations into actionable management measures, thereby fundamentally overcoming the common limitation of a disconnect between theory and practice in conservation planning [54,55]. Building on this framework, the study further proposes the establishment of an integrated conservation spatial pattern of one core area and two corridors (Fig 11), aiming to alleviate conflicts in resource use through optimized spatial organization and adaptive regulation, and ultimately to achieve the long-term coexistence and coordinated development of biodiversity and cultural diversity.

**Fig 11:**
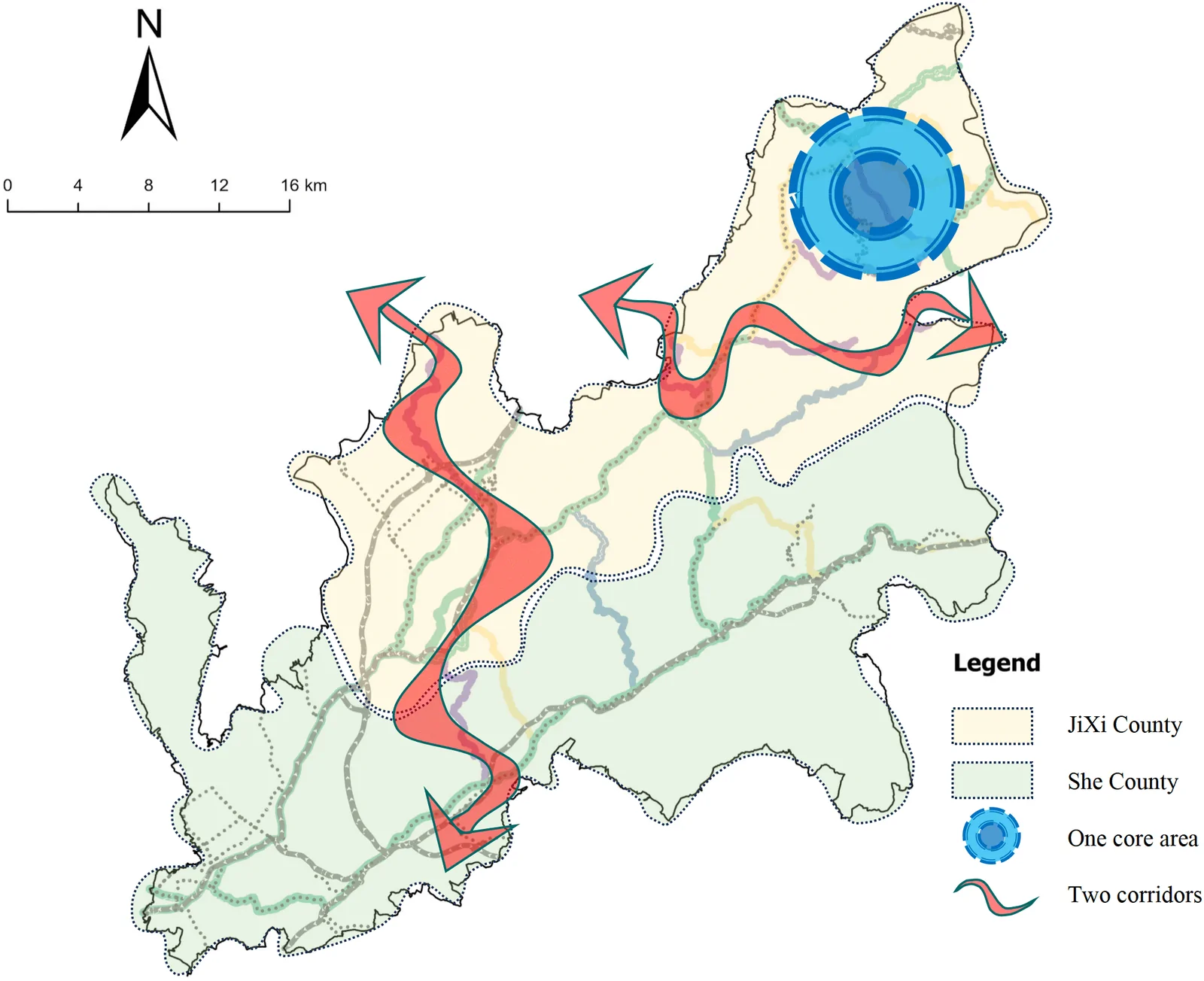
Landscape conservation pattern of the HHAT. HHAT: Huizhou-Hangzhou Ancient Trail. Note: This figure was created using map data from Tianditu, available at http://www.tianditu.gov.cn/.

*One Core Area*-Northeastern Jixi Ancient Trail Conservation Zone: This zone is anchored by the Wan-Zhe Sky Road and links six ancient trails (including two under strict conservation and four under restoration), forming a circular conservation spatial pattern. Within the zone, cultural heritage and ecological resources are moderately interwoven. Following the principle of priority to conservation and moderate revitalization, stringent measures are adopted to safeguard the integrity of the ancient trails and their surrounding habitats. Building on the existing base of self-driving tourism, the zone promotes low-impact cultural experience programs, such as Huizhou merchant-themed study tours, traditional farming experiences, and ancient trail hiking, supported by a digital reservation-based visitation platform and native vegetation-based ecological buffer zones [56].

*Two Corridors* are conceived as integrated conservation corridors addressing both natural and cultural dimensions. The two corridors follow differentiated planning pathways based on variations in resource endowments. The first corridor—the Xiaoyaoling AT–Dade AT–Yangxi–Fuling AT corridor—links the ecologically significant Xiaoyaoling AT with the Dade AT, which is rich in cultural heritage. Guided by a design concept integrating ecological and cultural experiences. this corridor establishes a continuous mobility route through adaptive modification of existing trails, thereby strengthening the connectivity and interaction between ecological and cultural resources. The second corridor—the Huanlianling AT–Huiling AT–Wangkengling AT corridor—largely retains its original landscape character and is underpinned by a high-quality natural environment. Adhering to the nature- and wilderness- focused design philosophy, only essential ecological and heritage restoration measures are implemented. The corridor prioritizes low-intensity hiking, exploration, and nature observation activities, thereby preserving the region’s native ecosystems and landscape integrity [57].

At the implementation level, the core approach is spatial-control zoning with differentiated strategies: Ecological core zones strictly limit all forms of anthropogenic intervention to maintain the integrity of native ecosystems. Cultural core zones focus on the adaptive utilization of heritage resources, while simultaneously promoting vegetation restoration and habitat improvement to achieve synergy between cultural continuity and ecological conservation. Transitional zones promote integrated ecology–culture development models, such as exploring pathways that combine understory economic activities with the transmission of intangible cultural heritage craftsmanship. Targeted strategies are formulated for the four conservation types. Conservation-Minimal Intervention prioritizes the preservation of the authenticity of ancient trails and their surrounding environments, applying only minimal intervention and strictly avoiding disturbance to native ecosystems. Conservation-Appropriate Revitalization allows carefully controlled development that does not compromise the original ecological conditions, thereby achieving coordination between conservation and revitalization. Restoration-Minimal Intervention is guided by the restoration of historical authenticity and employs minimally invasive restoration techniques. Restoration-Appropriate Revitalization, building on the completion of heritage restoration, emphasizes the transmission of cultural memory through the creation of distinctive cultural experience settings [43].

### 5.3 Study limitations and prospects

This study develops an integrated analytical and planning framework for linear cultural heritage landscapes from a biocultural diversity perspective and validates its applicability through the case of the HHAT. Nevertheless, several limitations remain. (1) The analysis is based on spatial data from a single snapshot and therefore does not capture the dynamic evolution of coupling relationships between biodiversity and cultural diversity; future studies could address this by incorporating multi-temporal remote sensing data and historical records. (2) The indicator system for biodiversity and cultural diversity is primarily spatial in nature, with relatively limited representation of ecological processes, functional diversity, and intangible cultural practices, which warrants further expansion. (3) Explanations of antagonistic coupling mechanisms are mainly grounded in macro-scale structural factors, and the relative contributions of different driving forces still require quantitative assessment. (4) The Marxan-based conservation schemes constitute scenario simulations, and their long-term effectiveness remains to be verified through practical implementation and adaptive management.

## 6 Conclusion

This study uses the HHAT as a case to reveal the characteristics of the spatial relationship between biodiversity and cultural diversity within mountainous linear cultural heritage landscapes from a biocultural diversity perspective. It explores and proposes different conservation approaches for the HHAT and corresponding spatial planning strategies. Results indicate a pronounced antagonistic coupling pattern between biodiversity and cultural diversity across the study area, with high-value zones exhibit spatial misalignment. This pattern may suggest potential influences of topographic constraints, concentrated human activities, and historical trail dependencies on the association between biological and cultural diversity. By integrating biocultural diversity indicators with the Marxan optimization model, this study achieves a spatial translation from relationship identification to conservation priority delineation, addressing gaps in decision support within traditional cultural heritage conservation research. The proposed differentiated conservation and spatial regulation strategies provide a reference pathway for refined management of mountainous linear cultural heritage landscapes under the dual context of ecological conservation and cultural heritage preservation.

## Supporting information

S1 File Minimal datas et (ZIP)

## Abbreviations

HHAT: Huizhou-Hangzhou ancient trail
BCD: Biocultural diversity index
BCCPA: Biocultural core protected areas
AT: Ancient trail
BD: Biodiversity index
